# TGR5 activation ameliorates hyperglycemia-induced cardiac hypertrophy in H9c2 cells

**DOI:** 10.1038/s41598-019-40002-0

**Published:** 2019-03-06

**Authors:** Kai-Chun Cheng, Wei-Ting Chang, Feng Yu Kuo, Zhih-Cherng Chen, Yingxiao Li, Juei-Tang Cheng

**Affiliations:** 1 Department of Psychosomatic Internal Medicine, Kagoshima University Graduate School of Medical and Dental Sciences, Kagoshima, 890 Japan; 2 Department of Cardiology, Chi-Mei Medical Center, Yong Kang, Tainan City, 71003 Taiwan; 3 Department of Medical Research, Chi-Mei Medical Center, Yong Kang, Tainan City, 71003 Taiwan; 4 Cardiovascular Center, Veterans General Hospital, Kaohsiung City, 81362 Taiwan; 5 Department of Pharmacy, Chia Nan University of Pharmacy & Science, Jean-Tae City, Tainan County, 71701 Taiwan

## Abstract

Left ventricular hypertrophy is an independent risk factor in diabetic patients. TGR5 is shown to express in hearts, but its functional role in diabetes-induced cardiac hypertrophy remained unclear. The current study investigated the role of TGR5 on high glucose-induced hypertrophy of H9C2 cells. After incubation with a high level of glucose, H9C2 cells showed hypertrophic responses. Activation of TGR5 by lithocholic acid (LCA) ameliorated cell hypertrophy and enhanced SERCA2a and phosphorylated phospholamban (PLN) expression in H9C2 cells. Triamterene inhibited these effects at an effective dose to block TGR5. However, LCA failed to modify the free radical elevation induced by high-glucose in the H9c2 cells. Moreover, PKA inhibitors, but not an Epac blocker, markedly improved hyperglycemia-induced hypertrophy and attenuated the increased SERCA2a expression by LCA; it also attenuated the phosphorylated PLN and SERCA2a protein expression levels in high glucose-treated H9C2 cells. In conclusion, TGR5 activation stimulated protein kinase A (PKA) to enhance PLN phosphorylation, which activated SERCA2a to remove Ca^2+^ from cytosol to sarcoplasmic reticulum in addition to the reduction of calcineurin/NFAT pathway signaling to ameliorate the hyperglycemia-induced cardiac hypertrophy shown in cardiomyocytes. TGR5 may service as a new target in the control of diabetic cardiomyopathy.

## Introduction

Bile acids (BAs) have been introduced as the byproducts of cholesterol metabolism in liver to secret into the duodenum^1^. Recently, BAs were also recognized as signaling molecules that may integrate with TGR5 or muscarinic receptors, the plasma membrane G-protein-coupled receptors, in addition to the nuclear receptors, including the farnesoid (FXR) and pregnane (PXR) xenobiotic receptors. The roles of BAs in regulating metabolic homeostasis and other important physiological functions have been documented^2,3^. BA binding sites and/or receptors are known to express in cardiovascular tissue, but the details regarding BA-induced changes in cardiovascular function are still unclear^4^.

TGR5, also named as M-BAR, BG37 or GPBAR1, is belonged to G-protein-coupled receptors (GPCRs). Therefore, TGR5 activation may induce cyclic AMP (cAMP) accumulation^5^. TGR5 expression has been identified in cardiomyocytes^6^. However, most observations were challenged to conduct the association between TGR5 and cardiac modulation without a direct effect^4^.

Cardiac hypertrophy, one of the initial disorders in cardiovascular system, is known to induce heart failure. Cardiac hypertrophy is usually identified by an increase in cell size including physiological and pathological hypertrophy^7^. Additionally, cardiac hypertrophy is also introduced as an elevation in protein synthesis and/or reactivation of the fetal gene program in cellular levels^8^. During the hypertrophic stimulation, calcineurinn dephosphorylated the nuclear factor of activated T-cells (NFAT) that may translocate into the nucleus to promote the gene expression, partly after forming a complex with GATA4. Therefore, calcineurin and NFAT are known for activation of the fetal gene program in response to hypertrophic stimuli, and they function as essential effectors during the formation of cardiac hypertrophy^9^. Consequently, atrial natriuretic peptide (ANP) and B-type natriuretic peptide (BNP) levels, which are raised as a result of hypertrophic gene expression, are used as clinical indicators^10^. Interestingly, ANP has shown antihypertrophic properties^11^. Moreover, the Ca^2+^ -calcineurin-NFAT signaling may integrate with another pathway, such as protein kinase C or mitogen-activated protein kinases (MAPKs), to coordinate the hypertrophic response^12^. Additionally, more transcription factors participated in cardiac hypertrophy were mentioned to explain it in detail^13^.

Diabetic cardiomyopathy (DCM) is one of the diabetic complication; cardiomyocytes exposed to high glucose levels exacerbates the hypertrophic response^14^. Many studies have used H9c2 cells to investigate hyperglycemia-induced cardiac damage^15,16^. However, the effect of TGR5 on DCM remains unknown^4^. Llithocholic acid (LCA), has been shown to modulate the bile acid pool and can specifically activate TGR5^17^. Thus, we used LCA to activate TGR5 and investigated the mechanism for alleviating the hyperglycemia-induced cardiac hypertrophy in cultured cardiac H9c2 cells. Additionally, cyclic AMP (cAMP) is the major cellular signal coupled to TGR5^5^. In the cAMP signaling pathway, protein kinase A (PKA) is activated by elevations in cAMP, and the exchange protein directly activated by cAMP (Epac) has been reported as another regulator of cAMP in the heart^18^. Therefore, we used specific inhibitors to investigate the potential mediation of LCA-induced effects in H9c2 cells by PKA or Epac.

## Results

### Lithocholic acid alleviates high glucose-induced cardiac hypertrophy in H9c2 cells

**Figure 1:**
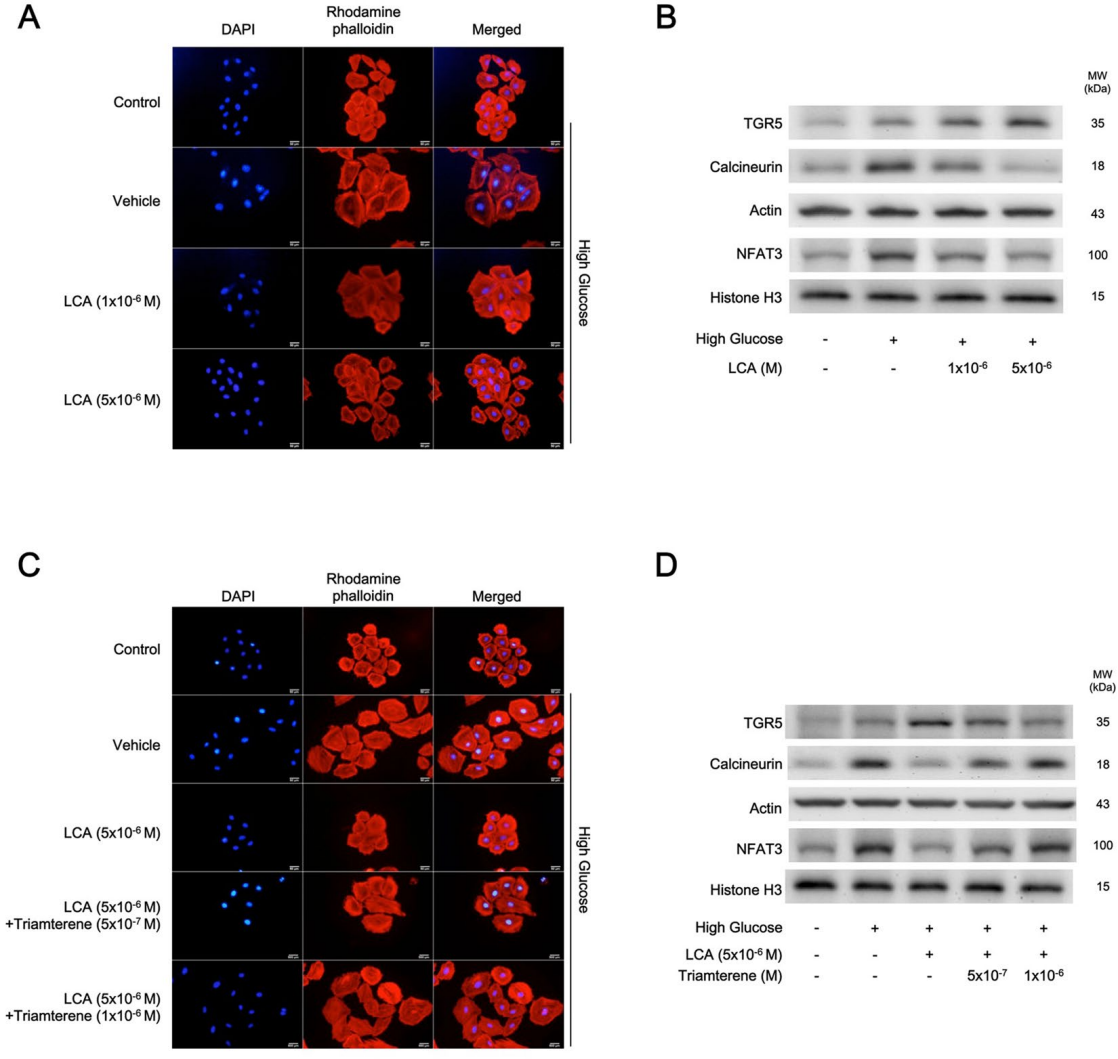
Effects of lithocholic acid (LCA) on high glucose-induced hypertrophy in H9c2 cells. (**A**) Morphological changes in H9c2 cells maintained in high-glucose medium (High Glucose) treated with vehicle (Vehicle) were compared with those of cells treated with LCA at the indicated doses or cells grown in normal medium (Control) (n = 6). (**B**) Western blots showing the TGR5 and hypertrophic signaling protein expression levels in H9c2 cells treated as described above and indicated in the representative image (n = 4). (**C**) The morphological changes in H9c2 cells under high glucose (High Glucose) conditions attenuated by LCA were reversed with triamterene at the indicated doses (n = 6). (**D**) Western blots showing the TGR5 and hypertrophic signaling protein levels in H9c2 cells treated as described in (**C**) are indicated in the representative image (n = 4). The quantified data are shown in Table 1.

In Fig. 1A, H9c2 cells exposed to high glucose (30 mmol/l) demonstrated a profound hypertrophic response. The mediation of osmolarity in the effects of high-glucose has been previously ruled out^19^. High-glucose treatment significantly increased in cardiomyocyte size compared to that of the normal group. Moreover, LCA inhibited high glucose-induced increases in cell size in a dose-dependent manner (Fig. 1A). Quantification of the changes in cell size is shown in Table 1. Additionally, changes in biomarker levels for cardiac hypertrophy were also assessed (Table 1); the results showed that ANP, BNP, and β-MHC mRNA levels changed in parallel. High glucose induced a marked upregulation of hypertrophy-associated signals, such as calcineurin and nuclear NFAT, as shown in Fig. 1B, and they were reversed by LCA treatment (Table 1). Although high-glucose increased the mRNA levels of hypertrophic biomarkers, LCA could attenuate them in parallel (Table 1). Interestingly, TGR5 protein expression was higher in hyperglycemic condition and it was further dose-dependently increased by LCA (Fig. 1B). Therefore, we identified that LCA may alleviate the hyperglycemia-induced cardiac hypertrophy using cultured cardiomyocytes.

**Table 1:** Effects of lithocholic acid (LCA) on the mRNA levels of hypertrophic biomarkers and intracellular calcium ions, in addition to the quantified data from Fig. 1A,B.

Parameters	Control	Vehicle in High Glucose	LCA (1 × 10^−6^M) in High Glucose	LCA (5 × 10^−6^M) in High Glucose
Cell size level (fold change) (n = 4)	1.00 ± 0.14	4.86 ± 0.18^**^	2.47 ± 0.15^*^^#^	0.98 ± 0.12^##^
Relative level of ANP/β-actin (n = 6)	1.00 ± 0.00	1.92 ± 0.05^**^	1.45 ± 0.06^*#^	1.01 ± 0.07^##^
Relative level of BNP/β-actin (n = 6)	1.00 ± 0.00	1.75 ± 0.07^**^	1.39 ± 0.04^*#^	1.11 ± 0.03^##^
Relative level of β-MHC/β-actin (n = 6)	1.00 ± 0.00	1.92 ± 0.05^**^	0.31 ± 0.05^*#^	0.92 ± 0.10^##^
[Ca2 + ](nM) (n = 6)	184.25 ± 6.23	216.00 ± 5.31^*^^*^	226.32 ± 2.02^*#^	186.94 ± 4.86^##^
Ratio of TGR5/β-actin protein (n = 4)	0.41 ± 0.05	0.57 ± 0.03^*^	0.69 ± 0.08^**#^	0.86 ± 0.10^**##^
Ratio of Calcineurin/β-actin protein (n = 4)	0.38 ± 0.04	0.80 ± 0.07^**^	0.60 ± 0.04^*#^	0.38 ± 0.06^##^
Ratio of NFAT3/Histone H3 protein (n = 4)	0.52 ± 0.06	0.91 ± 0.11^**^	0.69 ± 0.04^*#^	0.56 ± 0.03^##^

The changes in cell size shown in Fig. 1A were quantified for comparison in the first. The cell size was markedly enhanced by almost 5-fold by high glucose treatment (30 mM) in H9c2 cells and was reduced by lithocholic acid (LCA) treatment at the indicated doses. Similarly, the mRNA levels of hypertrophic markers, including ANP, BNP, and β-MHC, promoted by high glucose were also attenuated by LCA treatment in a dose-related manner. The merits of LCA for the amelioration of the hypertrophic response were supported by the changes in hypertrophic signals through calcineurin and NFAT3, as shown in the last two rows. Moreover, the intracellular calcium levels are indicated in the middle of the table. Each value is shown as the mean ± SEM at the indicated sample size (n) per group. *P < 0.05 and **P < 0.01 vs the control. ^#^P < 0.05 and ^##^P < 0.01 vs the vehicle-treated samples in high glucose (Vehicle in High Glucose).

### Triamterene inhibits the effect of lithocholic acid in H9c2 cells

To assess whether the effects of LCA were mediated by TGR5 in the cardiomyocyte, triamterene has been used as a TGR5 inhibitor^20^. In H9c2 cells, triamterene at doses sufficient attenuated the LCA-induced changes in cell size and hypertrophic signals (Fig. 1C,D), and the quantitative results were given in Table 2. Thus, the beneficial effects by LCA are mainly through an activation of TGR5 in H9c2 cells.

**Table 2:** Triamterene inhibited the effects of lithocholic acid (LCA) on the mRNA levels of hypertrophic biomarkers and intracellular calcium ions, in addition to the quantified data from Fig. 1C,D

Parameters	Control	Vehicle in High Glucose	LCA(5 × 10^−6^ M) in High Glucose	Triamterene(5 × 10^−7^M) + LCA(5 × 10^−6^ M) in High Glucose	Triamterene(1 × 10^−6^M) + LCA(5 × 10^−6^ M) in High Glucose
Cell size level (fold change) (n = 4)	1.00 ± 0.10	3.51 ± 0.22^**^	1.09 ± 0.14^##^	1.94 ± 0.13^*#^	3.81 ± 0.25^**^
Relative level of ANP/β-actin (n = 6)	1.00 ± 0.00	1.87 ± 0.07^**^	1.18 ± 0.09^##^	1.48 ± 0.04^*#^	1.94 ± 0.03^**^
Relative level of BNP/β-actin (n = 6)	1.00 ± 0.00	1.85 ± 0.05^**^	1.14 ± 0.05^##^	1.52 ± 0.04^*#^	1.75 ± 0.07^**^
Relative level of β-MHC/β-actin (n = 6)	1.00 ± 0.00	1.85 ± 0.03^**^	1.17 ± 0.05^##^	1.49 ± 0.07^*#^	1.93 ± 0.03^**^
[Ca2+](nM) (n = 6)	168.19 ± 4.65	247.83 ± 6.47^**^	175.03 ± 4.57^##^	199.33 ± 7.20^*#^	239.05 ± 8.16^**^
Ratio of TGR5/β-actin protein (n = 4)	0.28 ± 0.03	0.58 ± 0.05^*^	0.83 ± 0.07^**#^	0.70 ± 0.09^**#^	0.54 ± 0.02^*^
Ratio of Calcineurin/β-actin protein (n = 4)	0.33 ± 0.06	0.86 ± 0.03^**^	0.50 ± 0.04^##^	0.67 ± 0.03^*#^	0.84 ± 0.08^**^
Ratio of NFAT3/Histone H3 protein (n = 4)	0.41 ± 0.06	0.78 ± 0.08^**^	0.43 ± 0.08^##^	0.61 ± 0.05^*#^	0.76 ± 0.06^**^

The changes in cell size shown in Fig. 1C were quantified for comparison in the first row. The cell size enhanced by high glucose (30 mM) in the H9c2 cells was reduced by lithocholic acid (LCA) at the indicated dose, and this effect was reversed by triamterene in a dose-related manner. Similarly, the increased mRNA levels of hypertrophic markers, including ANP, BNP, and β-MHC, by high glucose that was attenuated by LCA were also reversed by triamterene. The merits of LCA for the amelioration of cellular hypertrophy, which was supported by changes in hypertrophic signals of calcineurin and NFAT3, were reversed by triamterene as shown in the last two rows. Moreover, similar changes in the calcium levels are indicated in the middle of the table. Each value is shown as the mean ± SEM at the indicated sample size (n) per group. *P < 0.05 and **P < 0.01 vs the control. ^#^P < 0.05 and ^##^P < 0.01 vs the vehicle-treated samples in high glucose (Vehicle in High Glucose).

High glucose treatment resulted in markedly increased calcium levels in H9c2 cells (Table 1). LCA significantly inhibited the increase in calcium levels (Table 1). As shown in Table 2, this action of LCA was also reversed by triamterene but it was not influenced by atropine at an effective dose to block muscarinic receptors. Intracellular Ca^2+^ levels were 178.3 ± 3.8 nM in the presence of atropine (1 μM), which was similar (P > 0.05) to the level in vehicle-treated samples (173.7 ± 7.0 nM) induced by LCA (10 μM) under high-glucose condition.

### Mediation of reactive oxygen species (ROS) in the effects of lithocholic acid in H9c2 cells

**Figure 2:**
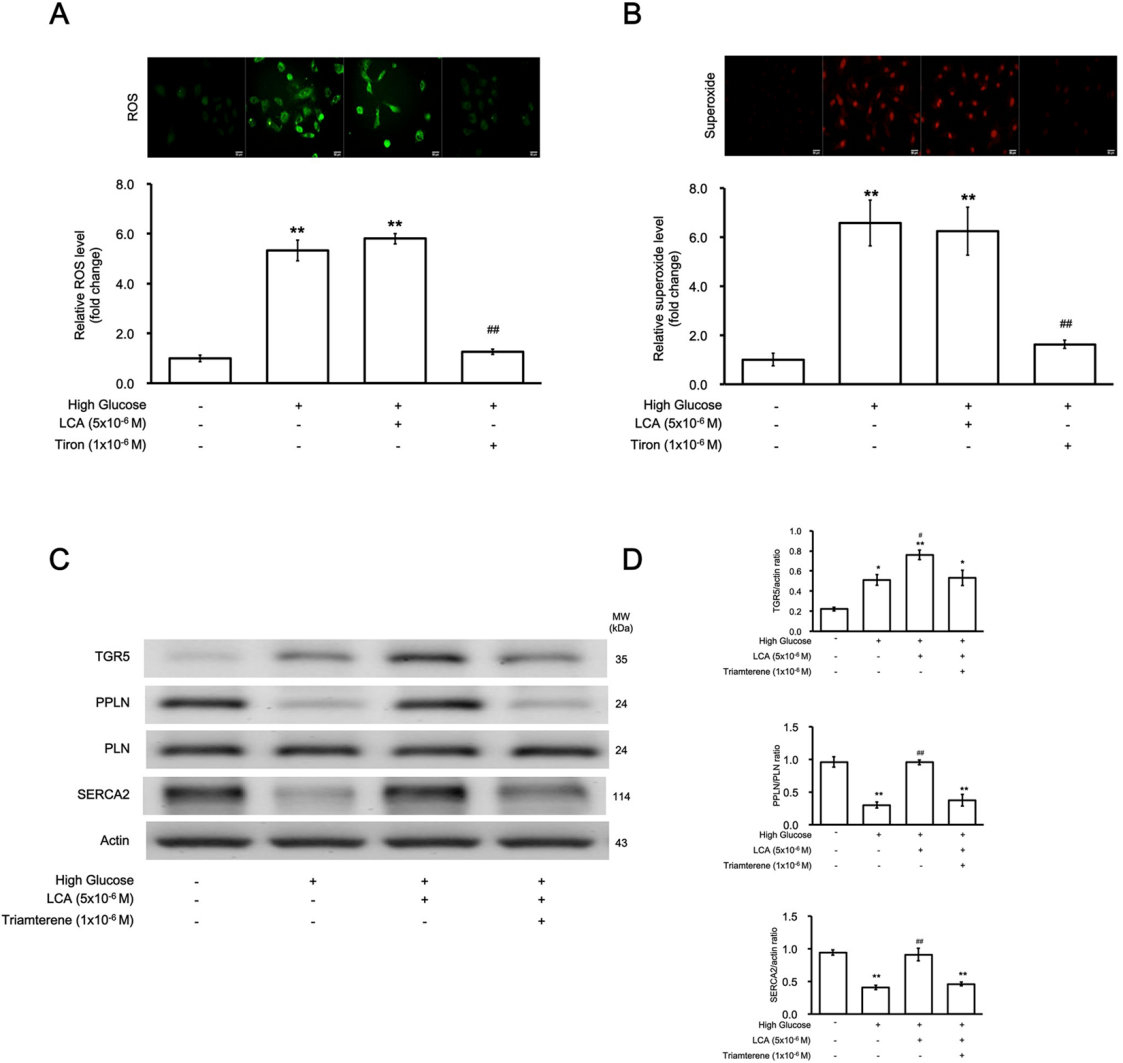
Effects of lithocholic acid (LCA) on oxidative stress-related signals in H9c2 cells with high glucose-induced hypertrophy. (**A**) Expression of ROS in H9c2 cells characterized using fluorescence as indicated in the upper panel. The quantified data from 6 experiments are compared in the lower panel (n = 6). (**B**) The ROS expression in H9c2 cells is characterized as described above. The results show that ROS elevated by high glucose was markedly reduced by the antioxidant tiron, but not by LCA (n = 6). (**C**) Representative image of Western blots indicating changes in protein levels of TGR5, SERCA2, phospholamban (PLN), and phosphorylated PLN (PPLN) using Actin as an internal control (n = 4). (**D**) The density of the different bands was quantified and compared (n = 4). The upper panel shows the variations in TGR5/Actin among the 4 groups, the middle panel compares the differences in PPLN/PLN expression among the 4 groups, and the lower panel shows the changes in SERCA2/Actin expression among the 4 groups (n = 4). The results from (**C**) and (**D**) suggest that the LCA-induced alleviation of the changes induced by high glucose was blocked by triamterene (*p-value < 0.05, **p-value < 0.01 versus control group; ^#^p-value < 0.05, ^##^p-value < 0.01 versus vehicle group).

As previously reported^14^, ROS induces DNA damage, which was markedly increased in H9c2 cells by high glucose (Fig. 2A). However, LCA treatment did not affect the increase in ROS, although the levels were markedly reduced by the antioxidant tiron.

### TGR5 activation prevents high glucose-mediated reduction of SERCA2a expression in H9c2 cells

As shown in Fig. 2, SERCA2a expression was markedly reduced in H9c2 cells under high-glucose conditions. This trend was markedly reversed by LCA treatment and was also blocked by triamterene. Thus, TGR5 activation ameliorates the reduction in SERCA2a expression in H9c2 cells under high glucose conditions. Additionally, LCA also promoted PLN phosphorylation in the same manner (Fig. 2D).

The SERCA2a mRNA level was also modified by LCA, increasing markedly to 1.18 ± 0.08 from 0.69 ± 0.36 under high-glucose conditions. The effect of LCA was blocked by triamterene; the mRNA level was only increased to 0.86 ± 0.14, which showed no difference with that under high-glucose conditions. However, the effect of LCA was not modified by the pre-treatment of GLP-1 receptor blocker exendin (9–39); the SERCA2a mRNA level promoted by LCA did not differ between the cells pretreated with exendin (9–39) and vehicle (0.97 ± 0.16 vs. 0.98 ± 0.17) under the high-glucose conditions. Therefore, mediation of GLP-1 receptor activation by LCA appears unlikely.

### Signaling coupled to TGR5 activation ameliorating high-glucose injury in H9c2 cells

**Figure 3:**
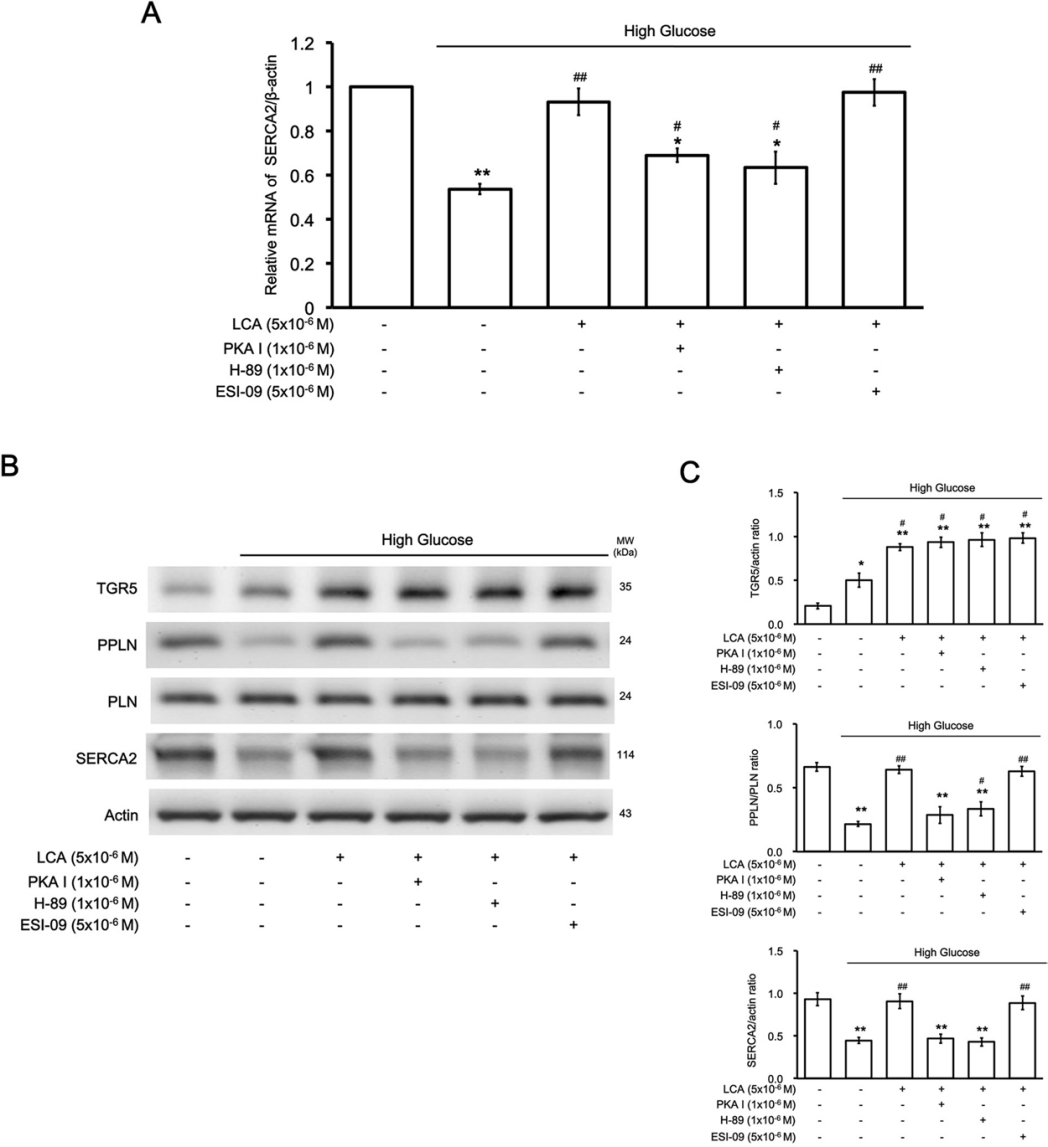
Identification of the signaling pathway responsible for lithocholic acid (LCA)-induced effects in H9c2 cells incubated in high-glucose medium. (**A**) Effects of LCA on the SERCA2 mRNA levels modified by high glucose in H9c2 cells were blocked by the protein kinase A (PKA) inhibitors PKAI and H-89, but not by the Epac inhibitor ESI-09 (n = 6). (**B**) Representative image of Western blots indicating the changes in protein levels of TGR5, SERCA2, phospholamban (PLN), and phosphorylated PLN (PPLN) using Actin as an internal control (n = 4). (**C**) The upper panel shows the variations in TGR5/Actin expression between the 6 groups, the middle panel compares the differences in PPLN/PLN expression between the 6 groups, and the lower panel shows changes in SERCA2/Actin expression between the 6 groups. The results indicate that cells treated with high glucose that improved with LCA treatment were blocked by the PKA inhibitors but not by the Epac inhibitor (n = 4), (* p-value < 0.05, **p-value < 0.01 versus control group; ^#^p-value < 0.05, ^##^p-value < 0.01 versus vehicle group).

Western blotting analysis shown in Fig. 3B indicated that LCA-promoted PLN phosphorylation was reduced by treatment with pharmacological inhibitors specific for PKA but not Epac. Additionally, PKA inhibition (via PKA I or H-89) and Epac inhibition (via ESI-09) failed to affect TGR5 expression in H9c2 cells treated with LCA. Consequently, recovery of the decrease in SERCA2a expression induced by LCA was similarly influenced. Taken together, our results show that TGR5 activation by LCA induces elevation of cAMP to activate PKA and increase PLN phosphorylation, which may relieve the inhibition of SERCA2a. Finally, the decrease in SERCA2a expression was reversed by LCA treatment.

### Effect of TGR5 activation on hypertrophy in H9c2 cells

**Figure 4:**
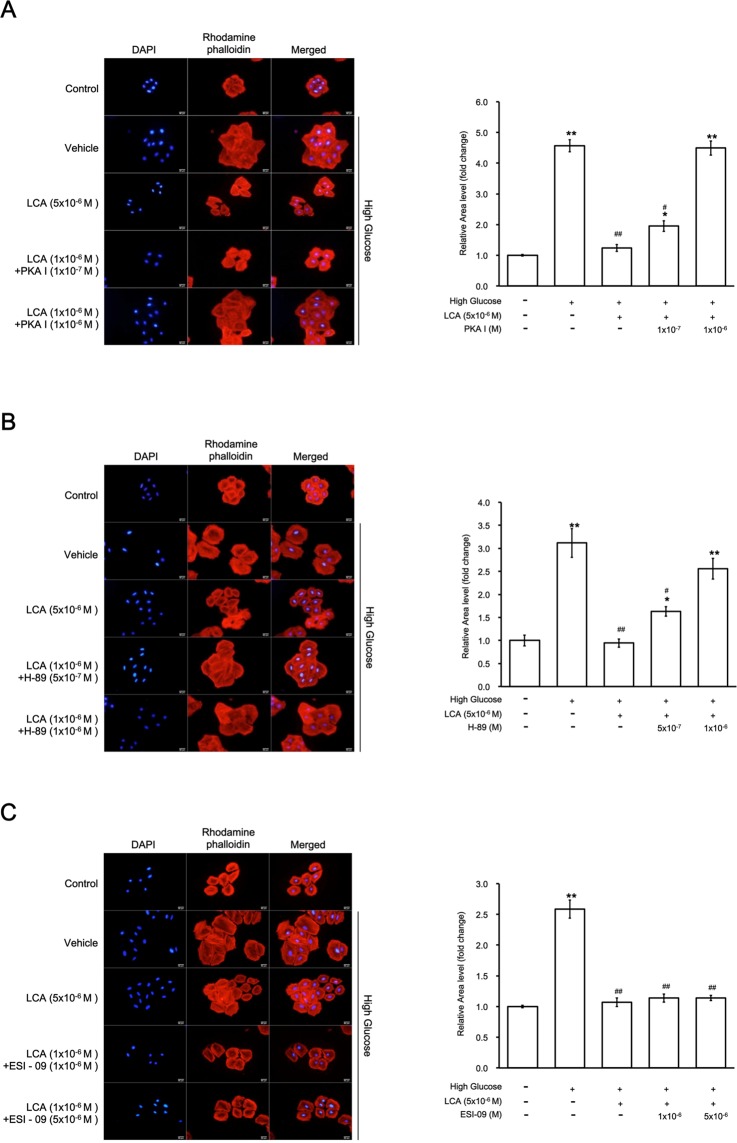
The effects of lithocholic acid (LCA) on hypertrophy are mainly mediated through protein kinase A (PKA) in H9c2 cells. (**A**) The effects of LCA were reversed by PKAI at the indicated doses; the right side shows the morphological changes in H9c2 cells, and the left side indicates the quantified data. (**B**) The effects of LCA treatment were also reversed by H-89 at the indicated doses; the right side shows the morphological changes in H9c2 cells, and the left side indicated the quantified data as described above. (**C**) Effects of LCA were not modified by ESI-09 treatment at doses effective to inhibit Epac; the right side shows the morphological changes in H9c2 cells, and the left side indicates the quantified data as above. Therefore, the LCA-induced alleviation of hypertrophy induced by high glucose is mainly mediated through the PKA pathway (*p-value < 0.05, **p-value < 0.01 versus control group; ^#^p-value < 0.05, ^##^p-value < 0.01 versus vehicle group, n = 6).

We utilized pharmacological inhibitors to identify major signaling pathways involved in H9c2 cell hypertrophy. As shown in Fig. 4, LCA alleviated hypertrophy in a manner that was sensitive to PKA inhibitors but not to Epac inhibitors (Fig. 4C). Therefore, linkage of PKA with TGR5 activation may ameliorate the high-glucose stimulated hypertrophic response in H9c2 cells.

## Discussion

The present study found that TGR5 activation might ameliorate hyperglycemia-induced hypertrophy in H9c2 cells. These novel results demonstrate the effectiveness of TGR5 in a relevant cell line. The presence of the TGR5 receptor in the heart has previously been demonstrated^6^. TGR5 activation has been reported to have cytoprotective effects in the mouse heart^21^. TGR5 belonged to G-protein-coupled receptor, also named as M-BAR, GPBAR1 or BG37, is an established target of bile acids. Therefore, TGR5 activation may result in cAMP accumulation and/or receptor internalization^5^. cAMP is introduced to stimulate cardiac function^22^, but mice with biliary fibrosis show cardiac dysfunction^6^. The influence of bile acids in heart seems likely due to an integration with TGR5 and muscarinic receptors, the plasma membrane G-protein-coupled receptors, in addition to the nuclear receptors, such as the FXR and PXR xenobiotic receptors^2,3^.

We used the rat-derived H9c2 cell line in current study to investigate the role of TGR5 activation according to previous report^23^. The cells were exposed to high glucose to mimic diabetic cardiomyopathy as described previously^15^. Similarly, hypertrophy was identified in H9c2 cells under hyperglycemic conditions and was induced through the calcineurin/NFAT hypertrophic pathway based on Western blotting analysis. Then, we used the established cell model to investigate the influence of TGR5 activation using LCA, which has previously been documented as a physiological ligand for TGR5^24^. Interestingly, LCA ameliorated the hypertrophy in H9c2 cells in a dose-dependent manner. The effects of LCA were reduced by triamterene treatment at a dose used to inhibit TGR5^20^. Therefore, TGR5 activation alleviated hyperglycemia-induced hypertrophy in a cell model. These novel findings were supported by Western blotting data, which showed changes in hypertrophy-associated signaling proteins, and by RT-PCR, which was used to evaluate mRNA levels of hypertrophic biomarkers.

Oxidative stress is mentioned to involve in hyperglycemia-induced cardiac injury^25^ and is related to the development of diabetic cardiomyopathy^26^. The ROS-induced oxidative stress in cardiac and vascular myocytes is mentioned to link with cardiac hypertrophy^27^. The diabetic cardiomyopathy is developed by oxidative stress mainly due to the elevated ROS production and/or decreased antioxidant defenses^28^. High-glucose produces a marked elevation in ROS, including oxygen free radicals, in H9c2 cells^29^. Moreover, TGR5 activation appears to exert several effects on ROS production^30^. Our data showed that LCA reduced oxidative stress in hypertrophic H9c2 cells, which was consistent with previous reports^27,28,31^. However, LCA failed to modulate elevated free radical levels, which were significantly attenuated by treatment with the antioxidant tiron in H9c2 cells. This finding is similar to the effects of GLP-1 receptor activation, which showed that Exendin-4 did not affect the ROS levels elevated by high-glucose in neonatal cardiomyocytes^32^. Thus, the effects are downstream of free radicals and are likely associated with endoplasmic reticulum stress. Moreover, apoptosis is popularly used indicate the hyperglycemia-induced cardiac damage^16,25,32^. However, hypertrophic and apoptotic signaling pathways are known to be regulated by ROS in cardiac myocytes^27^. Cells possessed various sensitivities to oxidative stress, which leads to myocyte-specific hypertrophy and apoptosis phenotypes. H_2_O_2_ has been shown to induce hypertrophy at a dose lower than that used to produce apoptosis in H9c2 cells^33^ and adult rat cardiac myocytes^34^. In addition to observations in clinical practice, cardiac hypertrophy has been identified in mice with excess bile acid levels^6^. Therefore, we focused on hypertrophic changes in cardiac cells.

Calcium ions are known as the key-regulator of cardiac hypertrophy^9,12^. Although cardiac transcription factors are involved in hypertrophic responses^13^, the Ca^2+^-calcineurin-NFAT cascade has been established as the main pathway responsible for cardiac hypertrophy^12^. In the present study, high glucose levels increased Ca^2+^ levels in cardiac myocytes^15,35^. LCA attenuated the increased Ca^2+^ levels in a dose-dependent manner, and triamterene treatment revered these effects at a dose enough to inhibit TGR5. However, atropine treatment did not modify the effectiveness of LCA in H9c2 cells. The muscarinic M2 receptor has been reported to mediate bile acid-induced protection of arrhythmia in the heart^36^. These results agreed with a recent report using neonatal murine cardiomyocytes^37^ showing that ursodeoxycholic acid (UDCA) is a specific agonist of TGR5 but not FXR or M2 receptors. Therefore, LCA may reduce intracellular Ca^2+^ levels in H9c2 cells primarily through TGR5 activation.

The mechanisms underlying the reduction in Ca^2+^ levels in H9c2 cells in response to TGR5 activation did not associate with a decrease in free radicals, as shown above. Diabetic cardiomyopathy is related with a reduction in sarco/endoplasmic reticulum Ca^2+^ ATPase-2a (SERCA2a) expression and/or activity^32^. A decrease in SERCA2a levels is known to correlate with a lower myocardial function in addition to the impaired force-frequency and diastolic responses that may appear during the heart failure^41^. The membrane protein PLN is known to bind to SERCA2a, and phosphorylation of PLN may reduce SERCA2a inhibition and enhance SERCA2a function^38^. In current study, SERCA2a expression is markedly decreased by high-glucose in H9c2 cells^32^. Similarly, phosphorylated PLN was reduced. Triamterene blocked the effects of LCA at a dose used to inhibit TGR5. Our findings are similar to previous results regarding the GLP-1 receptor activation in neonatal mouse cardiomyocytes^32^. However, exendin-(9–39), the specific antagonist of GLP-1 receptor, did not modulate the LCA-induced changes in SERCA2a mRNA levels, which indicated that direct stimulation of GLP-1 receptors by LCA appears unlikely to occur.

Phosphorylation of PLN has been suggested to be cAMP-dependent^39^. TGR5 is known to enhance the cAMP accumulation^5^. Thus, TGR5 activation may promote PLN phosphorylation, as has been characterized in the current study. The classical cAMP effectors, e.g., PKA, and the exchange protein directly activated by cAMP (Epac), have been indicated in heart tissue^38^. We were therefore using pharmacological inhibitors to investigate the potential mediation by PKA or Epac on LCA-induced effects in H9c2 cells. Two pharmacologic inhibitors of PKA attenuated the effectiveness of LCA, however, an Epac blocker failed to produce similar inhibition. Thus, the increase in PLN phosphorylation by TGR5 activation is likely primarily PKA-dependent, which is similar to the GLP-1 receptor activation in neonatal murine cardiomyocytes^32^. PKA activates PLN via phosphorylation at Ser16^39^. It is interesting that LCA enhanced both SERCA2a and phosphorylated PLN levels. However, administration of PKA inhibitors and an Epac inhibitor did not affect the activation of TGR5 induced by LCA, which implied that a TGR5-triggered PKA/cAMP signaling pathway appears unidirectional. SERCA2a removes Ca^2+^ from cytosolic fraction to the sarcoplasmic reticulum, which is consistent with the reduction in the cellular Ca^2+^ levels induced by TGR5 activation. In animals, SERCA2a overexpressed hearts show an enhanced contractility, while SERCA2a knock-out mice develop heart failure earlier than the wild-type mice after pressure overload^39^. However, treatment with a TGR5 agonist did not modify the contractility in cardiomyocytes^37^. SERCA2a activated by a TGR5 agonist appears largely responsible for removing Ca^2+^ to the sarcoplasmic reticulum, which may alleviate hypertrophic responses in H9c2 cells. Therefore, as shown in Fig. 4, PKA inhibitor treatment reversed the effectiveness of TGR5 activation based on amelioration of hypertrophy in H9c2 cells.

There are certain limitations to the present study. Application of an animal model of hyperglycemia, primary rat cardiomyocyte culture and genetic manipulation of TGR5 to further clarify the role of TGR5 in cardiac hypertrophy will be included in future work.

In summary, we report novel findings that TGR5 activation may ameliorate the hypertrophic response in cardiac cells. TGR5 activation may stimulate PKA to enhance the phosphorylation of PLN, which activates SERCA2a to remove Ca^2+^ from cytosolic fraction and reduce hypertrophic signaling, e.g., via the calcineurin/NFAT pathway, to alleviate hypertrophy in H9c2 cells.

## Materials and Methods

### Materials

Atropine and exendin (9–39) from Sigma-Aldrich (St. Louis, MO, USA) were prepared in solution using culture medium. Additionally, lithocholic acid and triamterene from same supplier were prepared in dimethyl sulfoxide (DMSO) as stock solutions. The PKA inhibitor 14–22 amide (PKA I) is myristoylated at the N-terminus, which enhances its cell permeability (Tocris Bioscience, Bristol, UK). H89 is a potent selective inhibitor of PKA (Sigma-Aldrich). ESI-09 (Sigma-Aldrich) has been shown to block EPAC activity and function. Other reagents from indicated suppliers were all of analytical grade.

### Cell culture and treatment

H9c2 cardiomyoblast cell line (BCRC No. 60096) was cultured in Dulbecco’s modified Eagle’s medium (DMEM) and Supplemented with 10% FBS. To generate the cellular model of cardiac hypertrophy, high-glucose medium containing 30 mmol/L glucose and 2% FBS H9c2 cells were used to incubate with H9c2 cells for 48 h after achieving 70% confluence. The D-glucose (Sigma) was dissolved in normal medium to prepare a medium with 30 mmol/L glucose. In preliminary experiments, the hypertrophic responses were compared between H9c2 cells and primary rat neonatal cardiomyocytes under high-glucose conditions using RT-PCR. The mRNA level of hypertrophic genes, such as BNP and β-MHC^40^, in H9c2 cells was not varied with that in primary rat neonatal cardiomyocytes. It is the same as a previous report that hypertrophic responses in H9C2 cell line were similar as primary neonatal cardiomyocyte cells *in vitro*^41^.

### Measurement of hypertrophy in cells

Cells seeded into a 24-well plate were starved in the serum-free medium for 4 h before the treatment with a TGR5 agonist (lithocholic acid) for 72 h. Following treatment, at room temperature, we fixed the cells with 4% paraformaldehyde for 15 min and permeabilized them with 0.5% Triton X-100 for another 5 min. The actin filaments in cells were characterized after staining with rhodamine phalloidin (Invitrogen, Carlsbad, CA, USA) for 20 min. Then, the nucleus was identified with 4–6-diamidino-2-phenylindole dihydrochloride (DAPI) (Abcam, Cambridge, MA, USA) after staining for 15 min. Cells were imaged under fluorescence microscope (IX71; Olympus, Tokyo, Japan; magnification, ×200) with an imaging system (DP2-BSW) from same supplier. We measured the myocytes that completely observed in the field. Changes in cell size were then quantified using NIH ImageJ software^42^.

### Identification of intracellular ROS levels

In the present study, dihydroethidium (DHE) (Thermo Fisher Scientific Inc., Rockford, IL, USA) is applied to detect the intracellular ROS levels. Similar to a previous report^43^, H9c2 cells were seeded at a density of 7.5 × 10^3^ cells/ml in 24-well plates to incubate overnight. The cells were starved for 4 h and then exposed to LCA at the effective doses for another 72 h. After treatment, cells were incubated with 10 μM of DHE for 30 min at 37 °C. They were then fixed and imaged as described previously^43^. Five images of each sample were randomly selected under a microscope, and the average was used for analysis. We compared the results shown as percentage of the ROS level in cells from the image analysis as described above^42^.

### Measurement of changes in intracellular calcium levels

We measured the intracellular calcium levels using fura-2, a fluorescent probe, as described previously^44^. Changes in fluorescence were recorded using a fluorescence spectrofluorometer (F-2000; Hitachi, Tokyo, Japan). The [Ca^2+^]i values were estimated according to our previous report^44^. Background autofluorescence obtained from the unloaded cells was subtracted from all measurements. Additionally, the inhibitor effectiveness was compared after a 30-min pretreatment.

### Nuclear extraction

Nuclear fractions were extracted by the application of CNMCS Compartmental Protein Extraction Kit (BioChain Institute, Inc., Hayward, CA, USA) following a previous report^45^.

### Real-time reverse transcription-polymerase chain reaction (RT-PCR)

Following our previous report^46^, we estimated the mRNA level of each signaling target gene in current study. Briefly, TRIzol reagent (Invitrogen, Carlsbad, CA, USA) is applied to extract the total RNA from cell lysates. Then, 200 ng of the total RNA was reverse-transcribed into cDNA using the random hexamer primers (Roche Diagnostics, Mannheim, Germany). All PCR experiments were performed using a LightCycler (Roche Diagnostics GmbH, Mannheim, Germany). The primers for ANP, BNP, β-myosin heavy chain (β-MHC), SERCA2a, and β-actin are as follows:

ANP F: 5′-CACAGATCTGATGGATTTCAAGA-3′;

ANP R: 5′-CCTCATCTTCTACCGGCATC-3′;

BNP F: 5′-GTCAGTCGCTTGGGCTGT-3′;

BNP R: 5′-CCAGAGCTGGGGAAAGAAG-3′;

β-MHC F: 5′-CATCCCCAATGAGACGAAGT-3′;

β-MHC R: 5′-GGGAAGCCCTTCCTACAGAT-3′;

SERCA2a F: 5′-ATTGTTCGAAGTCTGCCTTCTGTGG-3′;

SERCA2a R: 5′-CATAGGTTGATCCAGTTATGGTAAA-3′;

β-actin F: 5′-CTAAGGCCAACCGTGAAAAG-3′;

β-actin R: 5′-GCCTGGATGGCTACGTACA-3′.

### Western Blotting Analysis

Western blotting analysis was performed using our previous method^42^. Protein extract were loaded at equal amounts into SDS-PAGE and transferred to a polyvinylidene difluoride membrane. Western blots were preformed to probe for calcineurin (Sigma-Aldrich, St. Louis, MO, USA), NFAT3 (Thermo-Fisher Sci., Rockford, IL, USA), SERCA2a (Cell Signaling), phospholamban (PLN) (Cell Signaling), and phosphorylated PLN (P-PLN) (S16/T17; Cell Signaling) in addition to the internal standard, β-actin (Sigma-Aldrich, St. Louis, MO, USA) and Histone H3 (Santa Cruz, Dallas, TX, USA). Subsequently, membranes were washed with 1 × PBS-Tween and hybridized with appropriate secondary antibody (1:5000). Membranes were incubated with the ECL detection reagent and imaged using the Avegene imaging system (Avegene Life Science, Taipei, Taiwan). Densities of the bands were quantified using NIH ImageJ software and normalized to β-actin or Histone H3 expression^45^. This assay was replicated with 4 independent experiments.

### Statistical Analysis

Results shown as means ± SEM were calculated from the determined samples (n) in each group. Statistical analysis using two-way analysis of variance (ANOVA) followed by Tukey’s post hoc analysis was used to compare the differences. Then, p < 0.05 was considered significant.

## Supplementary information

Supplementary Information SREP-18-39317
