# Supplementary material for: TGR5 activation ameliorates hyperglycemia-induced cardiac hypertrophy in H9c2 cells
Source: Sci Rep. 2019 Mar 6;9:3633. doi: 10.1038/s41598-019-40002-0 (PMC6403401; doi:10.1038/s41598-019-40002-0)

**Supplementary Information**

**SREP-18-39317**

**TGR5 activation ameliorates hyperglycemia-induced cardiac hypertrophy in H9c2 cells**

Kai-Chun Cheng, Wei-Ting Chang, Feng Yu Kuo, Zhih-Cherng Chen, Yingxiao Li*, Juei-Tang Cheng*

**Supplementary Fig S1** The full-length blots used in Fig. 1B.

Fig1 B TGR5 Fig1B Calcineurin


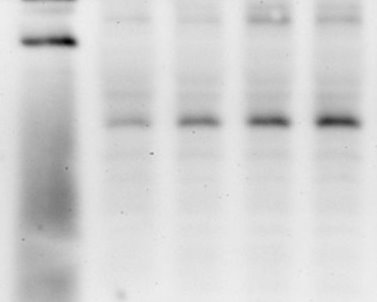

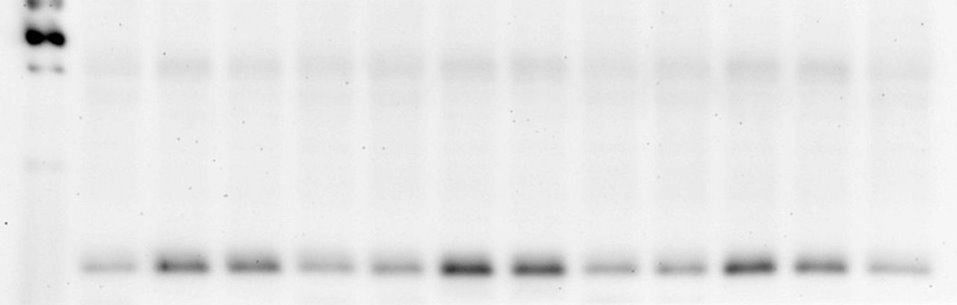


Fig1 B Actin Fig1B NFAT3


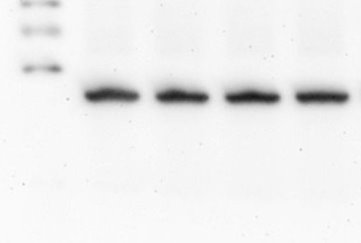

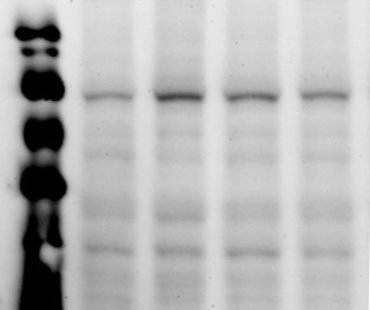


Fig1 B Histone H3


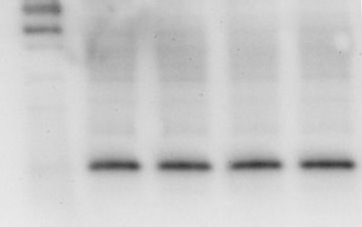


**Supplementary Fig S2** The full-length blots used in Fig. 1D.

Fig1 D TGR5 Fig1D Calcineurin


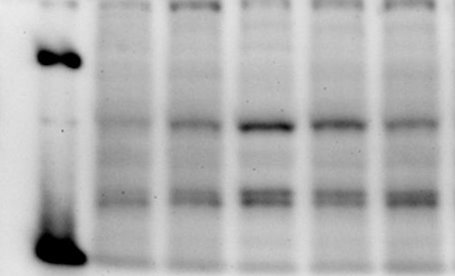

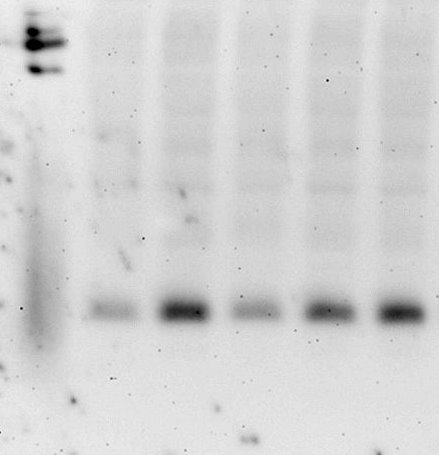


Fig1 D Actin Fig1D NFAT3


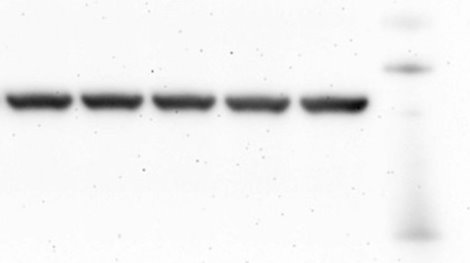

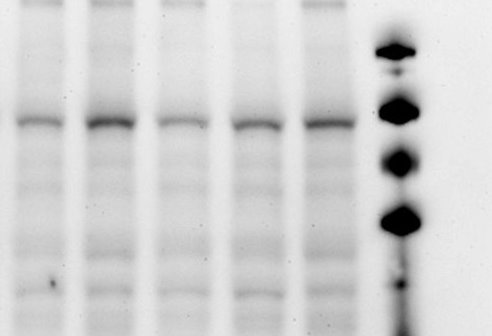


Fig1 D Histone H3


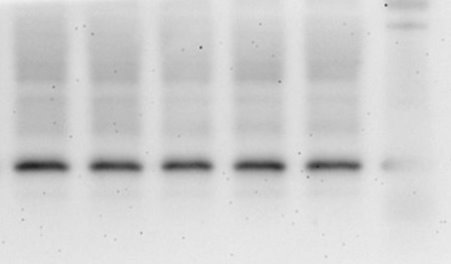


**Supplementary Fig S3** The full-length blots used in Fig. 2C.

Fig2C TGR5 Fig2C PPLN


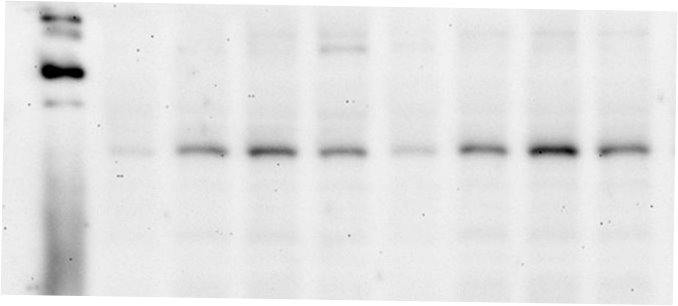

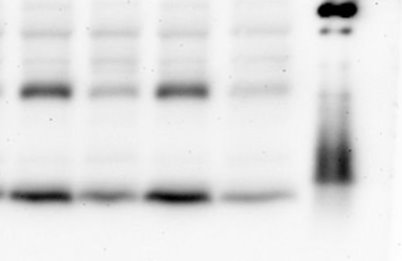


Fig2C PLN Fig2C SERCA2


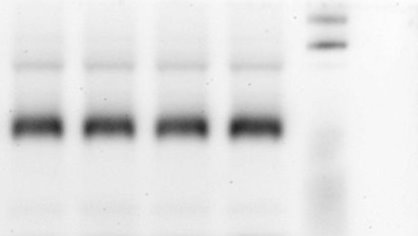

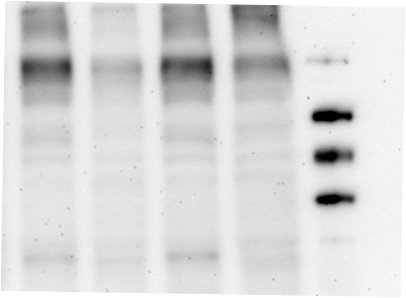


Fig2C Actin


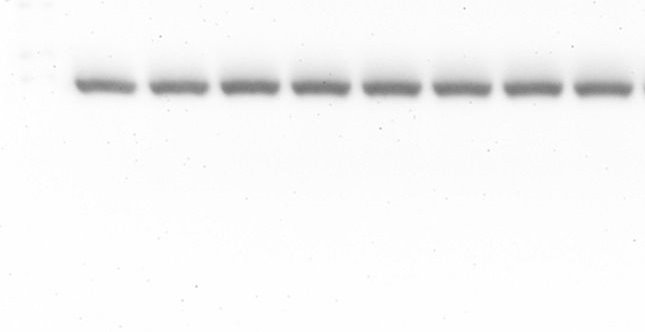


**Supplementary Fig S4** The full-length blots used in Fig. 3B.

Fig3B TGR5 Fig3B PPLN


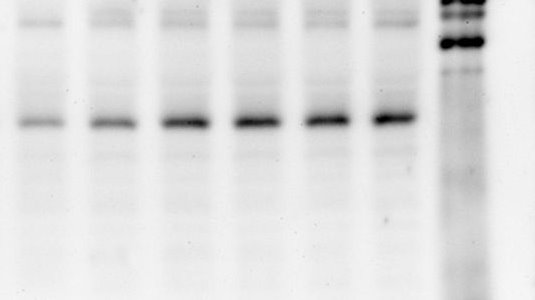

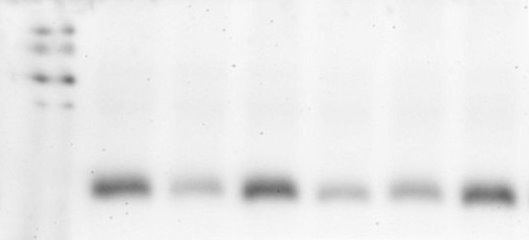


Fig3B PLN Fig3B SERCA2


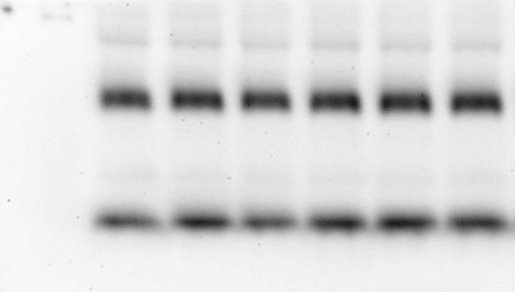

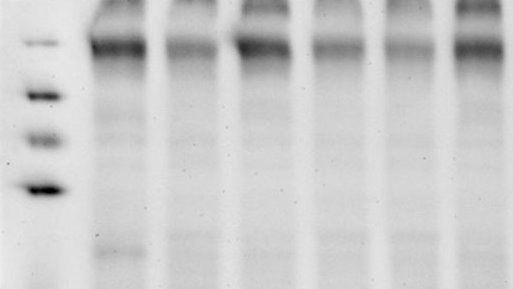


Fig3B Actin


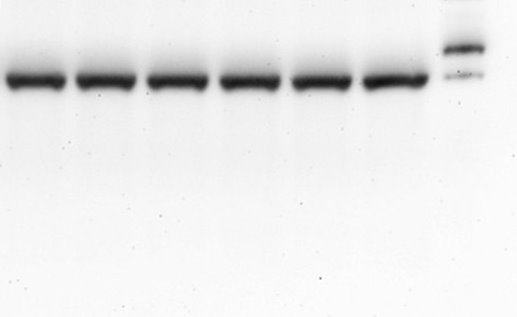

Supplement: Supplementary file 1 — Supplementary Information SREP-18-39317 [file 41598_2019_40002_MOESM1_ESM.doc]
